# Engineering Translation Components Improve Incorporation of Exotic Amino Acids

**DOI:** 10.3390/ijms20030522

**Published:** 2019-01-26

**Authors:** Takayuki Katoh, Hiroaki Suga

**Affiliations:** Department of Chemistry, Graduate School of Science, The University of Tokyo, 7-3-1 Hongo, Bunkyo-ku, Tokyo 113-0033, Japan

**Keywords:** EF-Tu, ribosome, tRNA, exotic amino acids

## Abstract

Methods of genetic code manipulation, such as nonsense codon suppression and genetic code reprogramming, have enabled the incorporation of various nonproteinogenic amino acids into the peptide nascent chain. However, the incorporation efficiency of such amino acids largely varies depending on their structural characteristics. For instance, l-α-amino acids with artificial, bulky side chains are poorer substrates for ribosomal incorporation into the nascent peptide chain, mainly owing to the lower affinity of their aminoacyl-tRNA toward elongation factor-thermo unstable (EF-Tu). Phosphorylated Ser and Tyr are also poorer substrates for the same reason; engineering EF-Tu has turned out to be effective in improving their incorporation efficiencies. On the other hand, exotic amino acids such as d-amino acids and β-amino acids are even poorer substrates owing to their low affinity to EF-Tu and poor compatibility to the ribosome active site. Moreover, their consecutive incorporation is extremely difficult. To solve these problems, the engineering of ribosomes and tRNAs has been executed, leading to successful but limited improvement of their incorporation efficiency. In this review, we comprehensively summarize recent attempts to engineer the translation systems, resulting in a significant improvement of the incorporation of exotic amino acids.

## 1. Introduction

In endogenous ribosomal translation, only the 20 proteinogenic amino acids (PAAs), the 19 l-α-amino acids, and glycine are available as building blocks for peptide/protein synthesis. On the other hand, the development of technologies for genetic code manipulation, such as nonsense codon suppression and genetic code reprogramming, has enabled the introduction of various nonproteinogenic amino acids (nPAAs) into the nascent peptide chain. l-α-amino acids with noncanonical side chains along with *N*-methyl-, d-α-, and β-amino acids, as well as α-hydroxy acid, have been successfully introduced [1,2,3,4,5,6]. Unfortunately, the efficiencies of ribosomal incorporation of nPAAs vary depending on their chemical structures, unlike that of PAAs [7]. For instance, nPAAs bearing nonproteinogenic bulky or phosphorylated side chains are generally poor substrates for translation. Moreover, the ribosomal incorporation of d-α- and β-amino acids is even more challenging, and their consecutive incorporation has been a formidable challenge, even after achieving single incorporation [4,5,8].

In fact, Fujino et al. reported in a 2013 paper that 19 d-α-amino acids were classified into three groups based on their single incorporation efficiency, and those classified in the top “efficient” group, such as d-Phe and d-Ala, could not be consecutively introduced into a model peptide [4,5,8]. They also successfully introduced 16 kinds of β-amino acids, but the most efficient members for single incorporation, such as β-homoglycine (β-hGly), l-β-homoalanine (l-β-hAla), l-β-homoglutamine (l-β-hGln), and l-β-homophenylglycine (l-β-hPhg), could not be consecutively introduced [4,5,8]. These results seemed to indicate a limitation of the translation system for the incorporation of such “exotic” amino acids. This has, in turn, encouraged researchers to further engineer the translation system and broaden its substrate tolerance.

**Figure 1:**
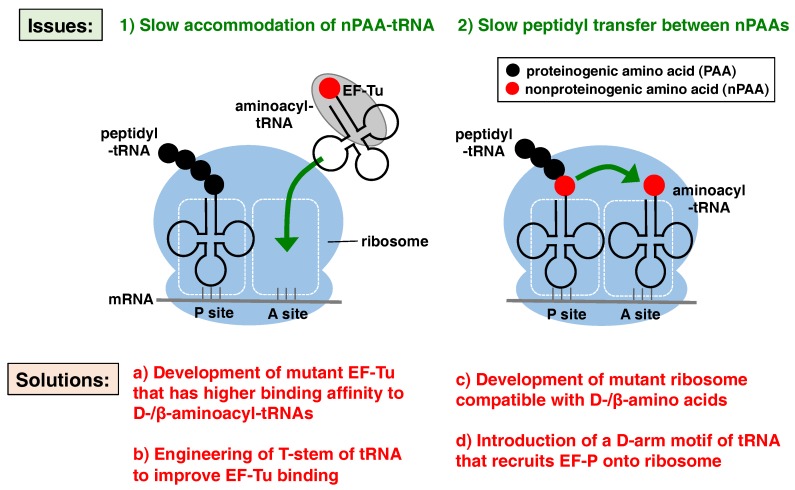
Reasons for the low incorporation efficiency of exotic nonproteinogenic amino acids (nPAAs). Green arrows indicate the slow accommodation of nPAA-tRNA (**1**) and slow peptidyl transfer between nPAAs (**2**), which are direct causes of low incorporation efficiency. Solutions to these issues for improving the incorporation of nPAAs (discussed in the main text) are listed in a–d. EF-Tu: elongation factor-thermo unstable; EF-P: elongation factor-P.

The low efficiencies for the incorporation of exotic amino acids could be attributed to the following two major reasons (Figure 1): (1) the slow accommodation of exotic nPAA-tRNA onto the ribosomal A site mediated by EF-Tu, and (2) the slow peptidyl transfer between the two exotic nPAAs in the ribosome catalytic center. Ribosomal stalling caused by slow accommodation and/or slow peptidyl transfer eventually induces mistranslocation of peptidyl-tRNA at the P site to the E site, resulting in the drop-off of the peptidyl-tRNA from the ribosome. Therefore, to improve the translation of peptides containing exotic nPAAs, the accelerated accommodation of nPAA-tRNA onto the ribosomal A site by EF-Tu, as well as the peptidyl transfer reaction between nPAAs, is necessary. Therefore, in this review, we summarize recent advances in the engineering of EF-Tu, ribosomes, and tRNAs to improve the accommodation and peptidyl transfer of nPAAs that lead to an improvement in the incorporation efficiency of nPAAs.

## 2. EF-Tu Engineering to Compensate for Poor Affinity to nPAA-tRNAs

**Figure 2:**
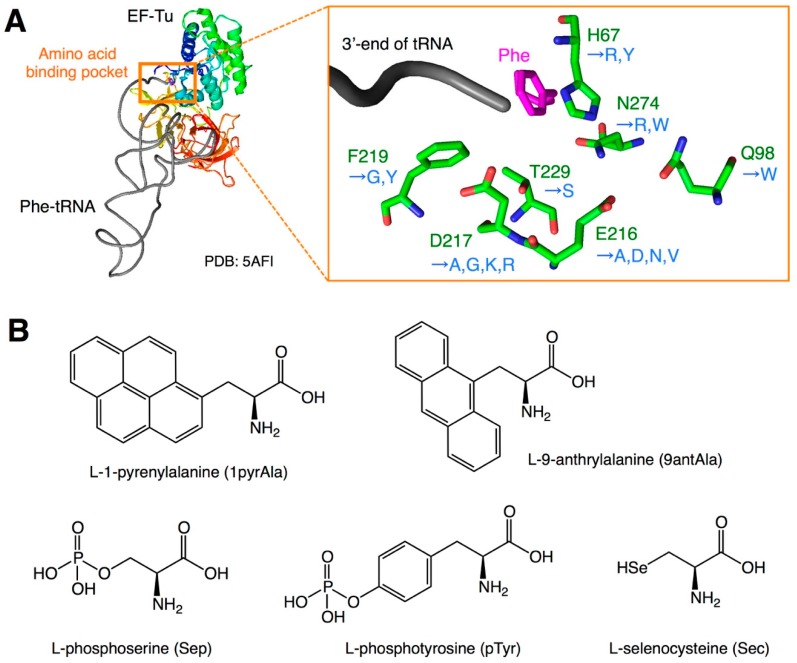
EF-Tu engineering to improve binding affinity toward exotic nPAA-tRNAs. (**A**) Residues of EF-Tu around the aminoacyl-tRNA binding pocket. Amino acids of the wild-type *Escherichia coli* EF-Tu are indicated in green, whereas mutations are indicated in blue. The 3′ end of phenylalanyl-tRNA bound to EF-Tu is shown, of which phenylalanine (Phe) is indicated in magenta, whereas tRNA is indicated in black. Structural information is taken from PDB: 5AFI. (**B**) Structures of amino acids used for ribosomal incorporation in combination with the engineered EF-Tu. Bulky a.a.: bulky amino acid.

The slow accommodation of nPAA-tRNAs could be because of the low binding affinity of nPAA-tRNAs toward EF-Tu. In addition, this insufficient binding increases the ratio of unbound nPAA-tRNAs, which are not protected by EF-Tu, and thus they are subjected to rapid ester-bond hydrolysis, making the incorporation of exotic nPAAs more inefficient. It has been previously reported that EF-Tu recognizes the amino acid moiety and T-stem region of aminoacyl-tRNAs [7,9]. For PAAs, although the binding affinity of EF-Tu toward the amino acid moiety varies depending on the amino acid structure, the overall binding affinity of EF-Tu toward aminoacyl-tRNAs is thermodynamically compensated for by the affinity of the T-stem region to have uniform affinities regardless of the type of amino acids [7,9]. For instance, amino acids with low affinity toward the amino acid binding pocket of EF-Tu, such as Glu and Asp, are charged onto tRNAs with a higher affinity toward EF-Tu (tRNA^Glu^ and tRNA^Asp^) [10,11]. Such binding affinity compensation is required to maintain uniform accommodation rates of various PAA-tRNAs onto the ribosome. However, the binding affinity of artificially designed nPAA-tRNAs is often much lower than that of PAA-tRNAs because of the extremely low binding affinity of the amino acid moiety, which is not compensated for by the inappropriate combination of tRNAs. For instance, exotic nPAAs with bulky or negatively charged side chains are less efficiently bound by EF-Tu. Therefore, to enhance the binding affinity between nPAA-tRNA and EF-Tu, various efforts have been made to develop EF-Tu mutants (Figure 2A, Table 1).

In a pioneer work by Doi et al. EF-Tu mutants that efficiently bind bulky amino acids, such as l-1-pyrenylalanine (1pyrAla) and l-9-anthrylalanine (9antAla), were developed [12]. As the sizes of the side chains of these amino acids are too large compared with PAAs, the amino acid binding pocket of EF-Tu should also be enlarged for efficient binding. The authors focused on E216 and D217, which reside at the binding pocket, and constructed single and double alanine substitution mutants (E216A, D217A, and E216A/D217A). In these mutants, the side chains of glutamic acid and/or aspartic acid were replaced by smaller side chains of alanine to enlarge the binding pocket. (In Figure 2A and Table 1, the numbering of EF-Tu amino acids differs from that of the original paper by Doi et al. [12], because of the standardization required when discussing the different numbering in various studies; therefore, the variant names E215A, D216A, and E215A/D216A in the original paper are referred to as E216A, D217A, and E216A/D217A mutations, respectively.) Indeed, these three mutants show higher affinity toward 1pyrAla-tRNA and 9antAla-tRNA than the wild-type EF-Tu. When applied to the incorporation of 1pyrAla and 9antAla into streptavidin using the CGGG four-base codon, the E216A and D217A mutants showed significant improvement in their incorporation efficiency compared with the wild-type EF-Tu. In contrast, the E216A/D217A double mutant failed even in the incorporation of phenylalanine, thus providing no translation product.

Phosphorylated amino acids, such as l-phosphoserine (Sep) and l-phosphotyrosine (pTyr), are often found in diverse natural proteins, and are known to play biologically important roles (Figure 2B). Therefore, the direct incorporation of those amino acids by genetic code manipulation is of significance. Park et al. developed a mutant EF-Tu, named EF-Sep, for improving the binding affinity of Sep-tRNA [13]. The authors chose six residues of EF-Tu (H67, D216, E217, F219, T229, and N274) around the binding pocket for randomization, and generated a mutant EF-Tu library for in vivo selection. Vectors bearing the random EF-Tu library were then transformed into *Escherichia coli* and subjected to selection, in which the clones that efficiently introduced Sep at the amber codon in the chloramphenicol acetyltransferase gene survived on LB plates containing chloramphenicol. Consequently, they obtained a mutant with H67R, E216N, D217G, F219Y, T229S, and N274W mutations. This mutant EF-Tu demonstrated a higher binding affinity toward Sep-tRNA^Cys^, resulting in a higher Sep incorporation efficiency than the wild-type counterpart. It is likely that the substitution of negatively charged side chains in E216 and D217 with uncharged N and G residues, respectively, contributes to the observed tighter binding of the negatively charged phosphate group on the Sep side chain toward the binding pocket. Subsequently, Lee et al. reported the development of an improved EF-Sep, named EF-Sep21, which has E216V instead of E216N, in addition to the other mutations, resulting in a four-fold higher chloramphenicol resistance than the original EF-Sep [14]. For the incorporation of pTyr, Fan et al. took a similar approach where mutations were introduced around the binding pocket [15]. The authors reported that a variant referred to as EF-pY, containing the E216V, D217G, and F219G mutations, showed an improved efficiency of pTyr incorporation.

Selenocysteine (Sec) is also considered an important amino acid, which is often found in natural proteins. In nature, Sec is introduced using a Sec-specific elongation factor (SelB). SelB requires the Sec insertion sequence (SECIS) located downstream of the UGA codon for Sec-tRNA accommodation [16]. On the contrary, methods that use EF-Tu mutants for the SelB/SECIS-independent Sec incorporation have also been developed. As Sec is negatively charged under physiological conditions, the binding affinity of Sec-tRNA to EF-Tu is expected to be weak. Therefore, Haruna et al. developed EF-Tu variants that efficiently bind Sec-tRNA [17]. To this end, they rationally designed EF-Tu mutants depending on the sequence of SelB. For instance, the variant EF-R1 has the H67Y, E216D, D217R, and N274R mutations; however, this mutant demonstrated cytotoxicity. The authors also performed a selection of EF-Tu mutants using a randomized EF-Tu library, and obtained a mutant EF-Sel1 containing the H67R, Q98W, E216N, D217K, and N274R mutations, which demonstrated good binding affinity without generating cell toxicity.

EF-Tu mutants reported thus far were only for nPAA with bulky or negatively charged side chains, i.e., phosphate or selenide ions. To date, however, it is unknown if such mutants with exotic nPAAs, such as d-α- and β-amino acids with negatively charged side chains, are possible. Nevertheless, the studies mentioned above have indicated that the engineering of EF-Tu improves the intrinsically poor affinity toward nPAAs, which can be applied to other exotic nPAAs.

## 3. Ribosome Engineering to Improve Single Incorporation of Exotic Amino Acids

**Figure 3:**
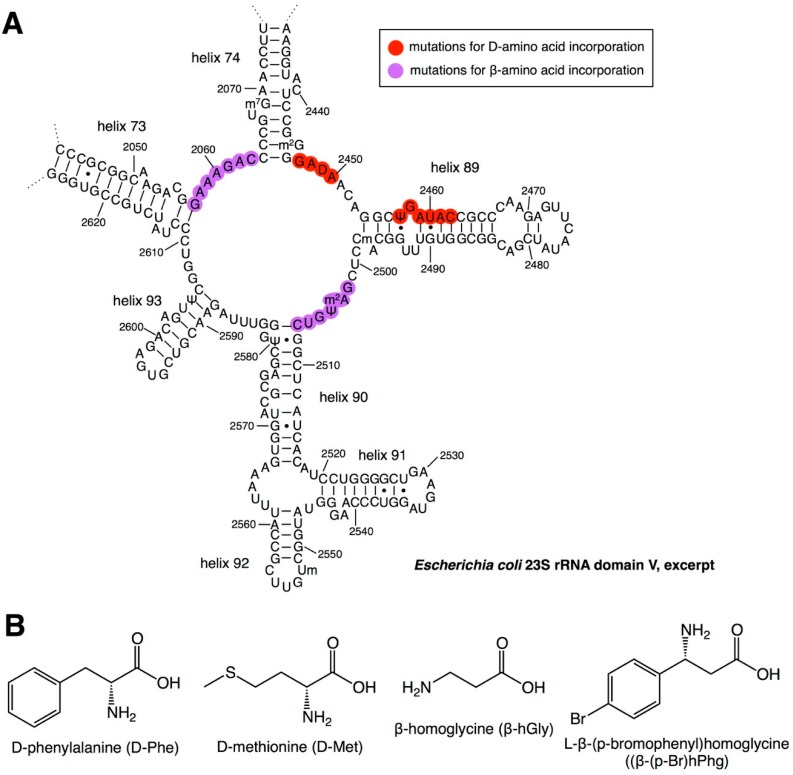
Engineering of ribosomal RNA for the incorporation of d- and β-amino acids. (**A**) Secondary structure of *Escherichia coli* 23S rRNA, domain V (excerpt). Positions of mutations introduced for d-amino acid incorporation are indicated by red circles, whereas those for β-amino acid incorporation are indicated by purple circles. (**B**) Structures of amino acids tested in translation using the engineered ribosome containing 23S rRNA mutations.

Although the peptidyl transferase center (PTC) of the ribosome can adapt to various side chains in PAAs and nPAAs, the incorporation of d-α- and β-amino acids is generally inefficient. This is likely because the amino nucleophile of these amino acids in the ribosomal A site cannot be appropriately positioned in the PTC to attack the carbonyl electrophile in the P site. To resolve this incompatibility, engineering efforts to introduce mutations in the PTC have been made. The catalytic activity of the peptidyl transfer reaction of the ribosome resides in domain V of the 23S rRNA (Figure 3A). For the incorporation of d-α-amino acids, Dedkova et al. constructed a series of mutant ribosomes containing mutations at 2447–2450 and 2457–2462 [18,19]. Among them, variants possessing 2447UGGC2450 and 2457GCUGAU2462 mutations (Table 2, variants A4 and B25) showed improved activity for introducing d-phenylalanine (d-Phe) and d-methionine (d-Met) (Figure 3B). Compared with the incorporation of l-Phe and l-Met as positive controls, the expression levels of the DHFR protein containing d-Phe and d-Met were 5.2% and 9.6% for the wild-type ribosome, and 22% and 49% for the mutant ribosome, respectively.

Similarly, the authors also developed ribosome mutants for efficient β-amino acid incorporation [20,21]. One variant that possessed the 2057AGCGUGA2063 and 2502UGGCAG2507 mutations (Table 2, variant 040329) exhibited an improved incorporation of β-hGly, β-hAla, β,β-dimethyl-β-hGly, β-hPhg, and β-(p-Br)hPhg (Figure 3B). Compared with the incorporation of l-α-Val as a positive control, the expression level of the DHFR with β-hGly was 4.0% and 12.3% for the wild-type and mutant ribosome, respectively. Based on their findings, Czekster et al. further optimized the sequence of these regions and obtained the variant P7A7, which has 2057AGCGUGA2063 and 2502UGACUU2507 mutations, resulting in a higher β-(p-Br)hPhg incorporation efficiency than the 040329 variant (Table 2) [22].

Despite the impressive results mentioned above, two issues should be pointed out. First, the mutant ribosomes reported in the present studies were unpurified for the activity assay, i.e., a crude S-30 extract or an in vivo system, in which the wild-type and mutant ribosomes co-exist. The contribution of the mutant ribosome to the incorporation of d-α- and β-amino acids could not be quantitatively estimated, because the ratios of the wild-type and mutant ribosomes included in these systems were undefined. Second, because of this, the intrinsic activity of mutant ribosomes toward PAAs is unknown, i.e., how poorly (or efficiently) the mutant ribosomes can translate peptides/proteins comprising only PAAs compared with wild-type ribosomes. Therefore, these experiments should be reproduced using a reconstituted translation system comprising purified mutant ribosomes for true estimation of mutant ribosome activity. Moreover, it is unclear if the consecutive incorporation of the d-α- and β-amino acids was examined, and what changes these mutations brought from a mechanistic point of view. Nevertheless, the above demonstration showed a possibility that PTC mutations on ribosomes allow the acceptance of exotic nPAAs.

## 4. tRNA Engineering to Improve Multiple and Consecutive Incorporations of Exotic Amino Acids

**Figure 4:**
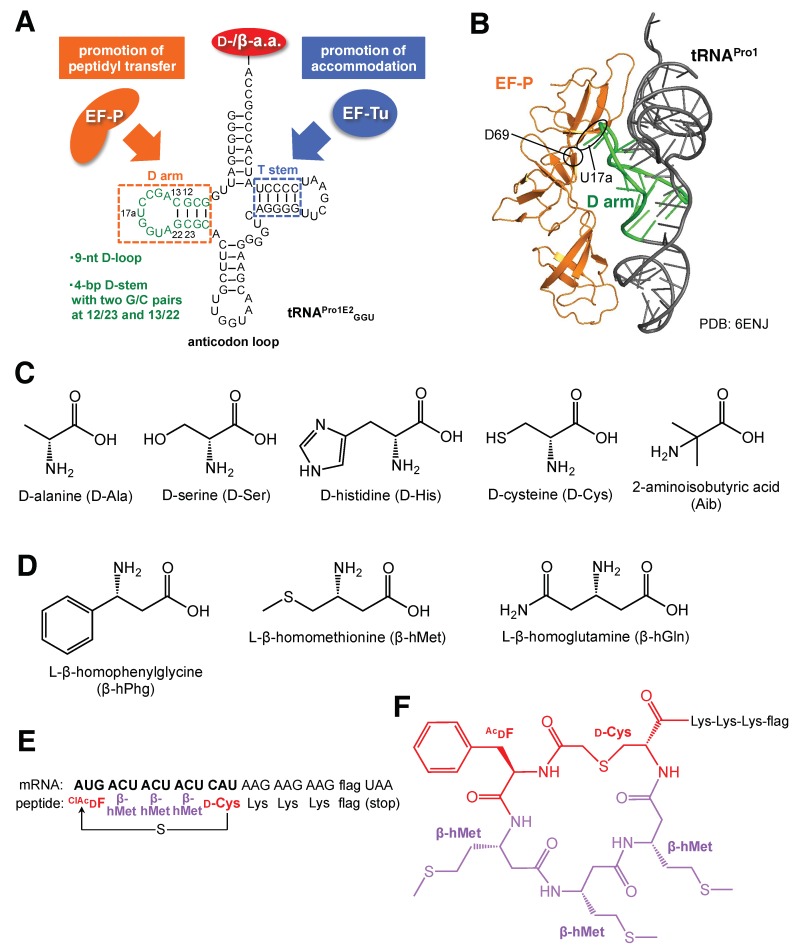
Incorporation of consecutive d- or β-amino acids by an engineered tRNA. (**A**) Secondary structure of tRNA^Pro1E2^_GGU_. The D-arm and T-stem motifs for tight EF-P and EF-Tu binding are indicated in orange and blue, respectively. The D-arm motif, consisting of a 9-nt D-loop closed by a 4-bp D-stem and containing two G/C pairs at positions 12/23 and 13/22, is shown. (**B**) Structure of *Escherichia coli* EF-P bound to P-site peptidyl-tRNA^Pro1^. Asp69 of EF-P and U17a of the tRNA D-loop are within hydrogen-bonding distance. Structural information is taken from PDB: 6ENJ. Structures of d- (**C**) and β-amino acids (**D**) tested for consecutive incorporation using tRNA^Pro1E2^ in combination with EF-P. (**E**) Sequence of a model peptide containing multiple d- and β-amino acids, and its template mRNA. The sulfhydryl group of the side chain of d-Cys spontaneously reacts with the chloroacetyl group of the N-terminal ^ClAc^dF to give a nonreducible thioether bond and macrocyclizes the peptide. (**F**) Structure of the model macrocyclic peptide, whose sequence is shown in E.

Despite the fact that the previously mentioned engineering of EF-Tus or ribosomes has not yet enabled the multiple incorporation of d-α- and β-amino acids in translation, a breakthrough occurred recently in tRNA engineering. Katoh et al. developed an engineered tRNA, named tRNA^Pro1E2^, bearing a chimeric body structure consisting of the T-stem motif of *E. coli* tRNA^Glu^ and the d-arm motif of *E. coli* tRNA^Pro1^ for tighter binding to EF-Tu and EF-P, respectively (Figure 4A) [23,24]. It should be noted that tRNA^Pro1E2^ was devised by a semilogical approach rather than an empirical approach. To compensate for the low affinity of d-α- and β-aminoacyl-tRNAs toward EF-Tu, the T-stem motif derived from tRNA^Glu^, for which the binding affinity to EF-Tu is high, was introduced to the designed tRNA. EF-P is a bacterial translation factor that accelerates peptide bond formation between consecutive Pro in the endogenous translation system [25,26]. EF-P recognizes the specific D-arm motif found in tRNA^Pro^ isoacceptors and binds to the P-site peptidyl-Pro-tRNA^Pro^ to prevent undesired peptidyl-Pro-tRNA drop-off. This results in the promotion of peptide bond formation between the P-site peptidyl-Pro-tRNA^Pro^ and the A-site Pro-tRNA^Pro^. We reported that the D-arm motif, consisting of a 9-nt D-loop closed by a stable 4-bp D-stem structure, contains two G/C base-pairs at positions 12/23 and 13/22 [27]. According to the cryo-EM structure of the EF-P/peptidyl-Pro-tRNA/ribosome complex, U17a in the D-loop of the P-site peptidyl-Pro-tRNA^Pro1^ is within hydrogen-bonding distance of the D69 backbone of EF-P (Figure 4B, PDB: 6ENJ) [28]. Such an interaction would not be possible for tRNAs with shorter D-loops, underscoring the importance of the conserved D-arm motif found in tRNA^Pro^ isoacceptors for EF-P binding. Using nPAA-tRNA^Pro1E2^, the chimera tRNA comprising the D-arm motif of tRNA^Pro^ and the T-stem motif of tRNA^Glu^, charged with d-α-amino acids (d-Ala, d-Ser, d-His, and d-Cys) and β-amino acids (β-hPhg, β-hMet, and β-hGln) by flexizymes, we demonstrated the consecutive elongation of these amino acids, which were significantly enhanced by adding EF-P to the translation system (Figure 4C,D) [23,24]. Moreover, an α,α-disubstituted nPAA, amino acid 2-aminoisobutyric acid (Aib), could be consecutively elongated (Figure 4C).

To further demonstrate the usefulness of this translation system, we also synthesized a macrocyclic peptide containing two d-α-amino acids and three β-amino acids, all of which were consecutively linked (Figure 4E,F). In this process, chloroacetyl-d-phenylalanine (^ClAc^dF), d-Cys, and β-hMet were pre-charged onto tRNA^fMet^_CAU_, tRNA^Pro1E2^_GUG_, and tRNA^Pro1E2^_GGU_ using flexizyme technology, and assigned to the initiator AUG, elongator CAU, and ACU codons of mRNA, respectively. It should be noted that the sequence of the anticodon loop of tRNA^Pro1E2^ can be arbitrarily chosen so that the precharged d- or β-amino acids are introduced at the corresponding codons of mRNA. Upon translation, the N-terminal chloroacetyl group of ^ClAc^dF spontaneously reacts with the side chain thiol of the downstream d-Cys, thus yielding a thioester macrocyclic peptide. The synthesis of this model peptide can be significantly enhanced by EF-P, indicating its acceleration effect; this was accomplished without any undesired misincorporation of other amino acids at the codons used for the incorporation of d-α- and β-amino acids.

Most importantly, tRNA engineering is simple compared to ribosome or EF-Tu engineering, and quite effective for achieving multiple and consecutive incorporation of some exotic amino acids. Some exotic amino acids are still difficult to incorporate consecutively, but the approach of a combination of tRNA^Pro1E2^ with ribosome mutations, for instance, would be beneficial to consecutive incorporation of more aggressive exotic amino acids.

## 5. Perspectives

Although the incorporation of nPAAs with bulky or negatively charged side chains was significantly improved by engineering the amino acid binding pocket of EF-Tu, improvement of d-α- and β-amino acid incorporation by engineered EF-Tu has not yet been reported. Achenbach et al. tested several EF-Tu mutants, such as E216A, E216G, E216S, N274A, and N274S (note that the mutant names in the original paper were E215A, E215G, E215S, N273A, and N273S, respectively), for d-α-aminoacyl-tRNA binding with an electrophoresis mobility shift assay, and confirmed that N274S showed a slightly better binding to d-α-aminoacyl-tRNA than the wild-type EF-Tu [8]. However, N274S showed no significant improvement in d-α-amino acid incorporation in translation. Nevertheless, considering that inefficient d-α- and β-amino acid incorporation is attributed to the low aminoacyl-tRNA binding of EF-Tu, more extensive screening of such EF-Tu mutants with higher binding affinities would be important in the future.

Although the studies reported to date have been focused on the engineering of EF-Tu among various translation factors, other translation factors, such as EF-G and EF4, would also be involved in regulating the incorporation of exotic amino acids. EF-G mediates the translocation of P-site deacylated tRNA and A-site peptidyl-tRNA into the E site and P site, respectively. However, when the P site and A site are occupied with peptidyl-nPAA-tRNA and nPAA-tRNA, respectively, because of their intrinsically slow peptidyl transfer reaction between the nPAAs, the ribosome would stall longer than usual. The accumulation of such a “stalling” state may lead to an increase in the frequency of peptidyl-nPAA-tRNA drop-off induced by EF-G, resulting in the truncation of the nascent peptide. Katoh et al. succeeded in improving the expression level of peptides containing consecutive d-α-amino acids by reducing EF-G concentration [29]. Conversely, EF4 mediates back-translocation of the stalled ribosome to resume translation. EF4 is not normally included in reconstituted translation systems, because it is not an essential factor for ribosomal translation. However, it would be worth testing EF4 for its ability to improve the incorporation of exotic amino acids. Although Achenbach et al. showed that EF4 did not improve nonconsecutive d-α-amino acid incorporation [8], EF4 may play a role in consecutive d-α- or β-amino acid incorporation with an appropriate tRNA structure such as EF-P [29]. To date, this issue has not been investigated.

In this review, we have not referred to the synthesis of nPAA-tRNAs; however, the selectivity and efficiency of the aminoacylation of nPAAs are also important topics for the incorporation of nPAAs. As natural aminoacyl-tRNA synthetases (ARS) cannot efficiently charge nPAAs onto tRNAs, alternative methods for preparing nPAA-tRNAs are necessary. Accordingly, the following methodologies have been developed so far: (1) the use of an aminoacylation ribozyme named flexizyme, which is used in the studies by Katoh et al. for the preparation of d-α- and β-aminoacyl-tRNAs [23,24,29,30,31], (2) charging nPAAs by means of mutant ARSs (used in the studies by Lee et al. and Fan et al.) [13,14,32,33,34,35,36], (3) the ligation of nPAA-pdCpA dinucleotides with tRNA lacking the 3′-terminal CA dinucleotide (used in the studies by Doi et al., Dedkova et al., and Maini et al.) [12,19,37,38,39], and (4) the post-aminoacylation modification of PAA-tRNA (used in the study by Haruna et al.) [1,17,40,41,42,43]. However, the details of these methodologies are discussed elsewhere [32].

Thanks to recent developments in engineered ribosomal translation systems, as discussed above, various nPAAs with unnatural side chains, d-stereochemistry, β-configuration, *N*-alkylation, and macrocyclic scaffolds have been successfully introduced into peptides and proteins to date. By applying such translation systems to decoding randomized mRNA sequences, peptide libraries containing various exotic amino acids with more than 1 × 10^12^ diversity can be simultaneously prepared within a 30 min translation time. The combination of ribosomally synthesized peptide libraries with display methods, such as mRNA display and ribosome display, enables the rapid discovery of bioactive peptides. Indeed, Suga et al. developed an mRNA display-based selection method, referred to as the Random nonstandard Peptides Integrated Discovery system, which enables the selection of peptides containing nPAAs using a reprogrammed genetic code with flexizyme-charged nPAA-tRNAs. Such systems provide a good drug-discovery platform for the development of structurally unique peptide ligands containing nPAAs [44,45,46,47,48,49,50,51,52,53,54,55,56,57,58,59,60,61].

## Figures and Tables

**Table 1:** List of engineered EF-Tu reported to date. Mutations introduced into EF-Tu and the amino acids tested for translation are shown. Note that the numbering of the amino acids of EF-Tu is derived from UniProtKB/Swiss-Prot: P0A6N3.2 (https://www.uniprot.org/uniprot/P0A6N3), and therefore some of the amino acid positions, indicated by asterisks, are different from the original paper.

Variant Name	Mutation	Amino Acid	Reference
E215A	E216A *	bulky a.a.	Doi et al. [12]
D216A	D217 *	bulky a.a.	Doi et al. [12]
EF-Sep	H67R, E216N, D217G, F219Y, T229S, N274W	Sep	Park et al. [13]
EF-Sep21	H67R, E216V, D217G, F219Y, T229S, N274W	Sep	Lee et al. [14]
EF-pY	E216V, D217G, F219G	pTyr	Fan et al. [15]
EF-R1	H67Y, E216D, D217R, N274R	Sec	Haruna et al. [17]
EF-Sel1	H67R, Q98W, E216N, D217K, N274R	Sec	Haruna et al. [17]

**Table 2:** List of the 23S rRNA mutants for d- and β-amino acid incorporation. Mutations introduced into 23S rRNA and the amino acids tested for translation are shown.

Variant Name	Mutation	Amino Acid	Reference
A4	2447UGGC2450	D-Phe, D-Met	Dedkova et al. [18]
B25	2457GCUGAU2462	D-Phe, D-Met	Dedkova et al. [19]
040329	2057AGCGUGA2063, 2502UGGCAG2507	β-hGly, β-hAla, β, β-dimethyl-β-hGly, β-hPhg, β-(p-Br)hPhg	Dedkova et al. [20] Maini et al. [21]
P7A7	2057AGCGUGA2063, 2502UGACUU2507	β-(p-Br)hPhg	Czekster et al. [22]

## Abbreviations

EF-Tu	Elongation factor-thermo unstable
EF-P	Elongation factor-P
EF-G	Elongation factor-G
EF4	Elongation factor 4
